# Challenges in Cranioplasty: A Case Report on Delayed Reconstruction and Postoperative Complications

**DOI:** 10.7759/cureus.80546

**Published:** 2025-03-13

**Authors:** Hussein Abourahma, Sayf Adas, Hiba Ahmad, Jessica Caushi, Tara Salimi, Tye Barber

**Affiliations:** 1 Internal Medicine, Nova Southeastern University Dr. Kiran C. Patel College of Osteopathic Medicine, Fort Lauderdale, USA; 2 Family Medicine, Nova Southeastern University Dr. Kiran C. Patel College of Osteopathic Medicine, Fort Lauderdale, USA; 3 Family Medicine, Broward Health Medical Center, Fort Lauderdale, USA; 4 Family Medicine, Broward Health Medical Center, Fort Lauderdale , USA

**Keywords:** autologous cranioplasty, cranioplasty timing, decompressive craniectomy, osteomyelitis, postoperative complications

## Abstract

Autologous cranioplasty is a crucial surgical intervention following decompressive craniectomy that is aimed at restoring cranial integrity and optimizing patient recovery. This report presents the case of a 59-year-old woman who experienced a substantial delay in cranioplasty due to unforeseen medical complications which contributed to the decision to defer treatment. The patient subsequently encountered a series of adverse events, including recurrent infections, neurological deficits, and bone flap resorption. This case underscores the importance of timely cranioplasty in minimizing complications and improving patient outcomes. Early reconstruction can enhance cerebral protection, improve neurological function, and reduce the risk of adverse events.

## Introduction

Decompressive craniectomy (DC) is a life-saving surgical procedure involving the removal of a portion of the skull to alleviate increased intracranial pressure. This procedure has become increasingly common in the management of traumatic brain injury, cerebral edema, and stroke [1]. Following DC, cranioplasty is performed to reconstruct the removed cranial segment, offering potential benefits such as cerebral protection, improved neurological function, and enhanced cosmetic appearance [2]. However, the optimal timing for cranioplasty remains a subject of debate [3].

The definition of early and late cranioplasty varies in the literature, making it quite challenging to accurately assess the risk of complications associated with delayed reconstruction. One study found that delays beyond three months may increase the risk of infection, hydrocephalus, hematoma formation, bone flap resorption, cognitive deficits, and cosmetic issues [4]. Further, early cranioplasty, performed within 30 days, may minimize bone flap resorption, infection, and seizures [5].

Despite these findings, the decision regarding when to perform cranioplasty is complex and influenced by patient-specific factors, including comorbidities, ongoing medical treatments, and personal preferences. In this report, we present a case of a 59-year-old woman whose cranioplasty was delayed for 17 months due to medical considerations and personal decisions, ultimately resulting in a challenging postoperative course. This case highlights the importance of individualized surgical planning, the potential risks associated with prolonged delays, and the role of a multidisciplinary approach in optimizing outcomes.

## Case presentation

**Figure 1:**
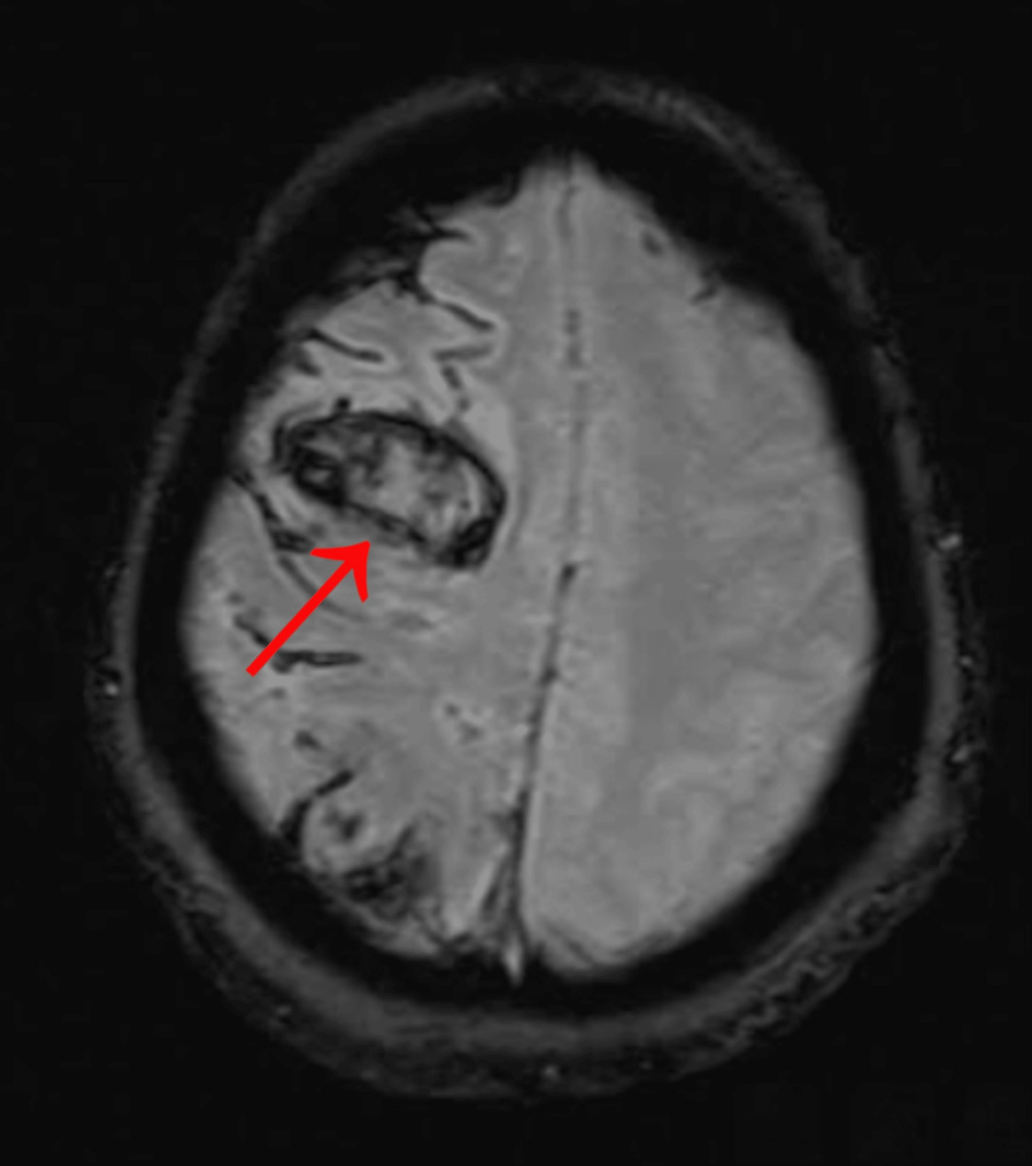
A Gradient Echo (GRE) MRI from August 2022 reveals a large right middle cerebral artery territory infarction, as indicated by the arrow.

A 59-year-old African American female patient initially presented to the emergency department (ED) with isolated acute left-sided weakness in August 2022, when imaging revealed a large right middle cerebral artery (MCA) territory infarct with hemorrhagic transformation (Figure 1). She underwent a right frontotemporoparietal decompressive craniectomy for malignant cerebral edema and subsequent evacuation of intracerebral hemorrhage. After a month of inpatient rehabilitation, she was discharged with a follow-up plan for a potential cranioplasty once the cerebral swelling had resolved. Unfortunately, approximately six weeks after being discharged, the patient presented to the ED with a pulmonary embolism (PE) and was discharged on anticoagulants. The neurosurgery team planned to perform a cranioplasty once the patient was weaned off the anticoagulants after three months. Although the patient had been counseled on the benefits of autologous cranioplasty, she declined and decided to postpone the surgery, further delaying the procedure. 

Approximately 17 months post the initial decompressive craniectomy, the patient elected to undergo a right frontotemporoparietal autologous cranioplasty. She was medically cleared and discharged three days postoperatively. However, the following month, she required antibiotics due to poor healing and areas of drainage along the incision. This marked the beginning of a prolonged course of complications, infections, antibiotic treatments, and multiple interventions. 

Two months later, despite prior treatments, the patient exhibited poor healing of her scalp which necessitated another course of antibiotics. Neurosurgery subsequently performed a right frontal scalp wound debridement and closure, retaining the bone flap while removing a burr hole cover.

**Figure 2:**
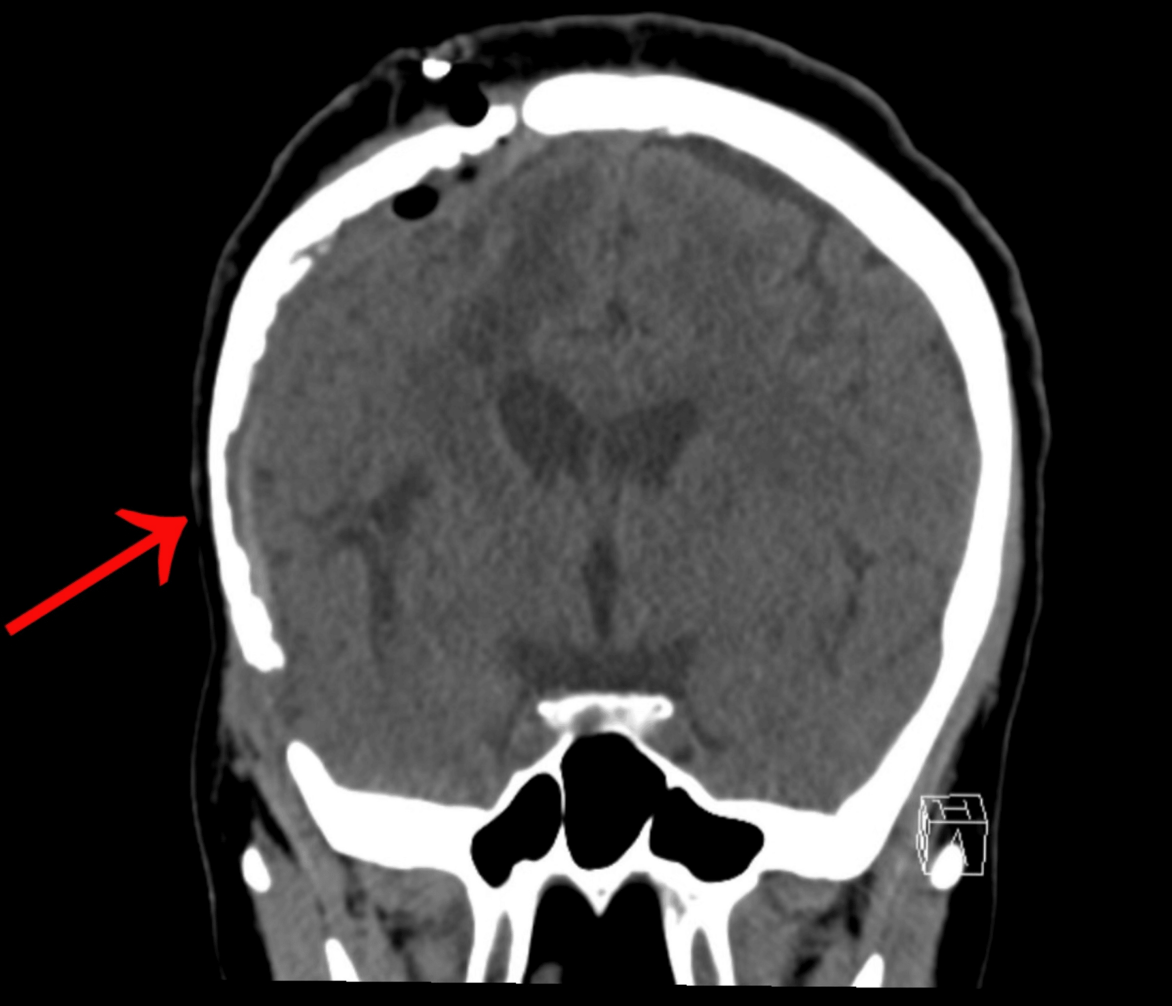
CT head without contrast completed August 2024. Right sided craniotomy with bone flap replacement. Bone flap demonstrating mottled appearance, in line with patient diagnosis of bone flap osteomyelitis, as indicated by the arrow. The epidural space deep to the flap is thickened with an intermediate density matrix, likely secondary to prior debridement the day prior.

By August 2024, two weeks post debridement, evaluation of the incision revealed small eschar and slight purulence. Cultures confirmed methicillin-resistant *Staphylococcus aureus* (MRSA), indicating chronic osteomyelitis of the bone flap (Figure 2). A subsequent craniectomy was required to remove the infected segment. The patient was discharged on intravenous (IV) vancomycin and oral rifampin, with ongoing follow-up to assess potential reconstruction options.

## Discussion

We presented the case of a 59-year-old female who received an autologous cranioplasty approximately 17 months post-decompressive craniectomy. There were numerous factors influencing the reason for the delay in this case. Initially, cranioplasty had to be delayed due to the patient starting anticoagulants after experiencing a PE approximately two months post-decompressive craniectomy. This increased the risk of compromised hemostasis and hematoma formation, putting her at risk for the procedure. Once the patient was weaned off anticoagulation, following a comprehensive discussion with neurosurgery regarding the risks and benefits of the procedure, the patient demonstrated an understanding of the information and elected to decline further surgery. 

Studies suggest that cranioplasty within 30 days may reduce infection and bone flap resorption rates, whereas procedures performed after 90 days may increase seizure risk but decrease hydrocephalus incidence [4,5]. Furthermore, cranioplasty was typically performed three months after decompressive craniectomy, as many neurosurgeons advocated for this time frame, to allow cerebral swelling to subside [6]. Therefore, unanswered questions remain regarding the optimal timing for cranioplasty and the physiologic and neurocognitive changes that accompany cranioplasty [7]. In this case, the significant delay in cranioplasty, influenced by the patient's need for anticoagulation and personal decision to postpone the procedure, may have contributed to a more complex clinical course. This ultimately necessitated three additional surgeries, during which the patient developed an infected bone flap requiring removal.

Beyond the physical challenges, delayed cranioplasty can also have a significant impact on a patient's psychological well-being. Prolonged hospitalizations, multiple surgeries, and ongoing medical interventions can lead to increased stress, anxiety, and depression. A holistic approach to patient care, including psychological support, is essential to address these challenges. Effective communication among healthcare providers is crucial to ensure optimal patient care. A multidisciplinary team approach, involving neurosurgeons, rehabilitation specialists, and primary care physicians, can help identify and address potential complications early on.

While this case highlights the potential risks associated with delayed cranioplasty, it is important to recognize that wound healing failure and infection are complications inherent to the procedure. This case report does not establish a direct causal link between delayed cranioplasty and increased morbidity, but it does contribute to the ongoing discussion regarding optimal timing for cranioplasty. Future studies should focus on standardizing definitions of early and late cranioplasty to enhance clarity in research and clinical practice.

## Conclusions

We present this case to underscore the significance of complications that may arise from prolonged delays in performing a cranioplasty. While different opinions exist on when to perform this procedure, excessive delays can lead to a more complicated recovery. In our case, the patient’s cranioplasty procedure was delayed 17 months, prompting three additional surgeries that ultimately led to an infected bone flap that necessitated removal. This series of complications highlights the potential for increased morbidity and the need for further interventions. This can significantly impact the quality of life and recovery of a patient. Therefore, a timely cranioplasty is essential to optimize patient recovery and decrease risks. While certain instances, like in this case, will force a delay in cranioplasty, we hope to highlight the importance of timely reconstruction, when possible.
